
JOURNAL INFORMATION
==============================
NLM Title Abbreviation: ScientificWorldJournal
Journal ID: ScientificWorldJournal
NLM Journal ID: 101131163
Journal ID: TSWJ

The Scientific World Journal

ISSN: 2356-6140
EISSN: 1537-744X
Publisher: Wiley

ARTICLE INFORMATION
==============================
PMCID: PMC5823190
PMID: 19618090
DOI: 10.1100/tsw.2009.87
Article ID: 801245
Article version: 1
Subjects: Directions in Science

Mysterious Tasks of Tyrosines in Syndecan-1 Cytoplasmic Tail

Rousselle Patricia *
Letourneur François
IFR128 BioSciences Gerland-Lyon Sud, Institut de Biologie et Chimie des Protés, Université de Lyon, 69367, Lyon, France
*Patricia Rousselle: p.rousselle@ibcp.frAcademic Editor: Martin Goette

Publication date: 2009
Electronic publication date: 2009 Jul 13
Volume: 9
First page: 629
Last page: 632
Received 2009 Apr 9; Revised 2009 Jun 26; Accepted 2009 Jun 29
Copyright: Copyright © 2009 Patricia Rousselle and François Letourneur.
Copyright year: 2009
License: This is an open access article distributed under the Creative Commons Attribution License, which permits unrestricted use, distribution, and reproduction in any medium, provided the original work is properly cited.
License URL: https://creativecommons.org/licenses/by/3.0/

Keywords: cell adhesion, syndecan, tyrosine phosphorylation, laminin 332, LG4/5 domain

Funding: Agence Nationale de la Recherche Ligue Contre le Cancer

==============================
Directions in Science
*TheScientificWorldJOURNAL* (2009) 9, 629–632
ISSN 1537-744X; DOI 10.1100/tsw.2009.87

TheScientificWorldJOURNAL
www.thescientificworld.com

### Mysterious Tasks of Tyrosines in Syndecan-1 Cytoplasmic Tail

Patricia Rousselle* and François Letourneur

*IFR128 BioSciences Gerland-Lyon Sud, Institut de Biologie et Chimie des Protéines, UMR 5086, CNRS, Université de Lyon, 7 passage du Vercors, 69367, Lyon, France*

**E-mail**: p.rousselle@ibcp.fr

*Received April 9, 2009; Revised June 26, 2009; Accepted June 29, 2009; Published July 13, 2009*

**KEYWORDS**: cell adhesion, syndecan, tyrosine phosphorylation, laminin 332, LG4/5 domain

Cell adhesion to the extracellular matrix (ECM) stimulates signal transduction cascades known to impinge on cell growth, differentiation, and cell death. Although integrins are the major cell surface receptors for the ECM, other adhesive systems, including transmembrane proteoglycans (such as syndecans), have recently drawn attention as an important class of adhesion receptors working in concert with integrins[1,2]. Like integrins, syndecans lack intrinsic enzymatic activities and thus transmit intracellular signals by interacting with various effector proteins, including both structural and signaling molecules. Following ligand binding, syndecans participate in the formation of adhesion complexes comprised of several actin-associated proteins that coordinate the "integration" of ECM components with the cellular cytoskeletal machinery. Cell-matrix adhesion receptors also activate kinases that phosphorylate signaling and cytoskeletal proteins, and thus regulate cell shape and motility[3].

Syndecans consist of transmembrane core proteins that carry either heparan sulfate (HS) or HS and chondroitin sulfate (CS) chains, through which they interact with a wide variety of molecules, including growth factors, cytokines, proteinases, adhesion receptors, and ECM components. There are four syndecans in mammals, which are expressed in a development-, cell-type–, and tissue-specific manner, and function either as coreceptors that cooperate with other cell surface receptors or as cell adhesion receptors that independently mediate cell signaling[4,5]. Among numerous ligands within the ECM, basement membrane laminins interact with these adhesion receptors to fulfill important cellular events[6]. Hence, the interaction between syndecan-1, predominantly expressed by epithelial cells, and a specific region in the laminin variant 332 (laminin 5, LN332) drives keratinocyte migration[7]. Furthermore, a cell adhesion model, in which syndecan-1 solely interacts with this isolated domain of LN332 (LG4/5 fragment recombinantly produced), has established that this interaction induces, in an integrin-independent manner, the formation of protrusive adhesion structures through activation of Rac and Cdc42 GTPases[7,8]. More recently, we reported that formation of these membrane protrusions requires dephosphorylation of tyrosine residues in the cytoplasmic tail of syndecan-1[9]. Indeed, cell adhesion to LG4/5 is remarkably associated with a rapid dephosphorylation of syndecan-1[9]. This observation was further supported by our results with genistein and staurosporine, two potent tyrosine kinase inhibitors previously shown to promote syndecan-1 down-phosphorylation[10], which significantly enhanced cell adhesion to LG4/5. These results unveil for the first time that one "turn on" signal for syndecan-1 upon LG4/5 recognition may not be phosphorylation, but tyrosine dephosphorylation, an unexpected outcome.

*Corresponding author.
©2009 with author.
Published by TheScientificWorld; www.thescientificworld.com

How this regulatory event takes place and which tyrosines are concerned appear now to be essential questions to address.

The syndecan-1 cytoplasmic domain is divided into two conserved regions, C1 and C2, which share common characteristics among all syndecans, and a central variable region known to regulate cell spreading and actin cytoskeleton assembly[11], as well as fascin bundling[12]. The C1 domain adjacent to the plasma membrane is thought to participate in syndecan dimerization[13] and in binding of various intracellular proteins, such as ezrin[5]. The conserved C2 carboxyl-terminal tetrapeptide sequence present in all syndecans, EFYA, binds some PDZ (postsynaptic density-95/disc large protein/zonula occludens-1) domain–containing proteins, such as syntenin-1[14] and CASK[15], which may function as membrane scaffold proteins that recruit signaling and cytoskeletal proteins to the plasma membrane. We have demonstrated that the PDZ-containing protein, syntenin-1, binds syndecan-1 in cells plated on the LG4/5 fragment and participates in the formation of membrane protrusions. Interestingly, its recruitment depends on the dephosphorylation of the tyrosine residue located within syndecan-1 PDZ binding domain EFYA[9]. Therefore, this step may be a key event in the molecular mechanism underlying tyrosine dephosphorylation of syndecan-1. Besides syntenin recruitment, tyrosine dephosphorylation of syndecan-1 may promote conformational changes of its cytoplasmic domain and/or provide binding sites for other proteins, and regulate syndecan-1 association with other cytoskeleton components. This hypothesis opens up new avenues of research.

Contrary to our syndecan-1 dephosphorylation signaling model, the regulation of syndecan-1 function by tyrosine phosphorylation was previously proposed[10], but no real link to functional features was ever proved. The syndecan-1 cytoplasmic domain contains four tyrosine residues that are conserved among all syndecan family members (Y1, Y2, Y3, Y4), among which Y1 is located within the transmembrane domain[16]. According to predictive analysis, two of the cytoplasmic tyrosine residues are likely targets for phosphorylation based on their surrounding amino-acid sequences (NetPhos 2.0 Server, www.cbs.dtu.dk/services/NetPhos). One of them (Y2) is located within the membrane-proximal conserved region C1 (DEGSY), and the other (Y4) is located within a second conserved sequence (EFYA) at the extreme carboxyl-terminal region C2. The third cytoplasmic tyrosine (Y3) is located within the V region. A study in which various domains of the syndecan-3 core protein, the closest homologue of syndecan-1, were expressed as fusion proteins in an *E. coli* cell line engineered to express the catalytic domain of *elk* kinase revealed that the tyrosine residues in the cytoplasmic domain can serve as substrates for phosphorylation by a tyrosine kinase[17]. However, it should be stressed that *in vivo* membrane lipids could impede tyrosine phosphorylation of the membrane proximal tyrosine (Y1). Phosphorylation at tyrosine residues Y2 and Y3 by the EphB2 receptor-tyrosine kinase was detected in the syndecan-2 cytoplasmic domain and was linked to dendritic-spine formation in neurons[18]. Whether one or several tyrosine residues in the syndecan-1 cytoplasmic tail are targets for phosphorylation *in vivo* and which kinases are involved is still unknown. The c-Src kinase might be a good candidate, as it was initially shown to bind the C1 region in syndecan-3[19] and its interaction with the syndecan-1 cytoplasmic tail was recently reported[20]. Interestingly, syndecan-1 phosphorylation appears to promote the cleavage of its ectodomain[10,21] and protein tyrosine kinases have been suggested to play a pivotal role in this shedding mechanism[22]. Conversely, a recent report challenged this hypothesis by showing that if tyrosines are surely involved, syndecan-1 shedding is not dependent on their phosphorylation state, but rather on the direct interaction with the small GTPase Rab5 and its subsequent activation through GDP-GTP exchange[20].

Participation of syndecan-1 in cytoskeleton dynamics and cell movement through integrin activation is a subject of particular interest at the moment. A regulatory mechanism of $\alpha V\beta 3$ and $\alpha V\beta 5$ integrins was identified through lateral interaction of ectodomains[23]. More recently, the syndecan-1 cytoplasmic tail was shown to associate with the $\beta 1$ integrin subunit[20], an association that may possibly be implicated in LN332-LG4/5 induced keratinocyte migration. Although integrins are not involved in the direct cellular interaction with the LG4/5 domain[7,8], their participation may occur via their connection with syndecan-1. Whether syndecan-1 tyrosines participate in these interactions or in other related molecular events is not yet elucidated. Such analysis would help to elucidate the dual, but probably sequential, participation of the tyrosines in syndecan-1 shedding vs. cell adhesion. Finally, the identification of the protein tyrosine phosphatase (PTP) associated to syndecan-1 signaling might be determinant to better understand these processes. Whether the phosphatase involved in this process belongs to the receptor-like PTPs or to the nontransmembrane phosphatase subfamilies remains a challenging question. Although experimental evidence has not been provided so far, some PTPs hold biochemical characteristics that grade them as potential candidates. Hence, the involvement of a receptor-like PTP would suggest that an interaction of its extracellular domain with a ligand may occur to form a complex and activate the intracellular PTP domain. An example among these is the receptor "leukocyte common antigen-related" protein (LAR), since syndecan-1 was recently described as being a ligand for LAR[24]. In addition, LAR was shown to bind LN-111 and play a role in regulating LN-111–induced cytoskeletal reorganization during cell spreading[25]. In conclusion, whether phosphorylation and/or dephosphorylation of tyrosine residues in syndecan-1 are important regulatory mechanisms of syndecan-1 function remains a crucial question, and additional work will definitively be needed to clarify this issue.

### ACKNOWLEDGMENTS

Original work by the authors was financially supported by the Agence Nationale pour la Recherche grant Chemispike, the Ligue Nationale contre le cancer, and the Association pour la Recherche Contre le Cancer.

### REFERENCES

1. Morgan, M.R., Humphries, M.J., and Bass, M.D. (2007) Synergistic control of cell adhesion by integrins and syndecans. *Nat. Rev. Mol. Cell Biol.* **8**, 957–969.
2. Streuli, C. and Akhtar, N. (2009) Signal co-operation between integrins and other receptor systems. *Biochem. J.* **418**, 491–506.
3. Legate, K.R., Wickström, S.A., and Fässler, R. (2009) Genetic and cell biological analysis of integrin outside-in signaling. *Genes Dev.* **23**, 397–418.
4. Tkachenko, E., Rhodes, J.M., and Simons, M. (2005) Syndecans: new kids on the signaling block. *Circ. Res.* **96**, 488–500.
5. Couchman, J.R. (2003) Syndecans: proteoglycan regulators of cell-surface microdomains? *Nat. Rev. Mol. Cell Biol.* **4**, 926–937.
6. Suzuki, N., Yokoyama, F., and Nomizu, M. (2005) Functional sites in the laminin alpha chains. *Connect. Tissue Res.* **46**, 142–152.
7. Bachy, S., Letourneur, F., and Rousselle, P. (2008) Syndecan-1 interaction with the LG4/5 domain in laminin-332 is essential for keratinocyte migration. *J. Cell Physiol.* **214**, 238–249.
8. Okamoto, O., Bachy, S., Odenthal, U., Bernaud, J., Rigal, D., Lortat-Jacob, H., Smyth, N., and Rousselle, P. (2003) Normal human keratinocytes bind to the alpha3LG4/5 domain of unprocessed laminin-5 through the receptor syndecan-1. *J. Biol. Chem.* **278**, 44168–44177.
9. Sulka, B., Lortat-Jacob, H., Terreux, R., Letourneur, F., and Rousselle, P. (2009) Tyrosine dephosphorylation of the syndecan-1 PDZ binding domain regulates syntenin-1 recruitment. *J. Biol. Chem.* **284**, 10659–10671.
10. Ott, V.L. and Rapraeger, A.C. (1998) Tyrosine phosphorylation of syndecan-1 and -4 cytoplasmic domains in adherent B82 fibroblasts. *J. Biol. Chem.* **273**, 35291–35298.
11. Carey, D.J., Bendt, K.M., and Stahl, R.C. (1996) The cytoplasmic domain of syndecan-1 is required for cytoskeleton association but not detergent insolubility. Identification of essential cytoplasmic domain residues. *J. Biol. Chem.* **271**, 15253–15260.
12. Chakravarti, R., Sapountzi, V., and Adams, J.C. (2005) Functional role of syndecan-1 cytoplasmic V region in lamellipodial spreading, actin bundling, and cell migration. *Mol. Biol. Cell* **16**, 3678–3691.
13. Oh, E.S., Woods, A., and Couchman, J.R. (1997) Multimerization of the cytoplasmic domain of syndecan-4 is required for its ability to activate protein kinase C. *J. Biol. Chem.*, **272**, 11805–11811.
14. Grootjans, J.J., Zimmermann, P., Reekmans, G., Smets, A., Degeest, G., Durr, J., and David, G. (1997) Syntenin, a PDZ protein that binds syndecan cytoplasmic domains. *Proc. Natl. Acad. Sci. U. S. A.* **94**, 13683–13688.
15. Cohen, A.R., Woods, D.F., Marfatia, S.M., Walther, Z., Chishti, A.H., and Anderson, J.M. (1998) Human CASK/LIN-2 binds syndecan-2 and protein 4.1 and localizes to the basolateral membrane of epithelial cells. *J. Cell Biol.* **142**, 129–138.

16. Yoneda, A. and Couchman, J.R. (2003) Regulation of cytoskeletal organization by syndecan transmembrane proteoglycans. *Matrix Biol*. **22**, 25–33.
17. Asundi, V.K. and Carey, D.J. (1997) Phosphorylation of recombinant N-syndecan (syndecan-3) core protein. *Biochem. Biophys. Res. Commun*. **240**, 502–506.
18. Ethell, I.M., Irie, F., Kalo, M.S., Couchman, J.R., Pasquale, E.B., and Yamaguchi, Y. (2001) EphB/syndecan-2 signaling in dendritic spine morphogenesis. *Neuron* **31,** 1001–1013.
19. Kinnunen, T., Kaksonen, M., Saarinen, J., Kalkkinen, N., Peng, H.B., and Rauvala, H. (2000) Cortactin-Src kinase signaling pathway is involved in N-syndecan-dependent neurite outgrowth. *J. Cell Biol.* **148**, 811–824.
20. Hayashida, K., Stahl, P.D., and Park, P.W. (2008) Syndecan-1 ectodomain shedding is regulated by the small GTPase Rab5. *J. Biol. Chem*. **283**, 35435–35444.
21. Reiland, J., Ott, V.L., Lebakken, C.S., Yeaman, C., McCarthy, J., and Rapraeger, A.C. (1996) Pervanadate activation of intracellular kinases leads to tyrosine phosphorylation and shedding of syndecan-1. *Biochem. J*. **319**, 39–47.
22. Fitzgerald, M.L., Wang, Z., Park, P.W., Murphy, G., and Bernfield, M. (1998) Shedding of syndecan-1 and -4 ectodomains is regulated by multiple signaling pathways and mediated by a TIMP-3-sensitive metalloproteinase. *J. Biol. Chem*. **273,** 10702–10708.
23. Beauvais, D.M., Ell, B.J., McWhorter, A.R., and Rapraeger, A.C. (2009) Syndecan-1 regulates alphavbeta3 and alphavbeta5 integrin activation during angiogenesis and is blocked by synstatin, a novel peptide inhibitor. *J. Exp. Med*. **206,** 691–705.
24. Fox, A.N. and Zinn, K. (2005) The heparan sulfate proteoglycan syndecan is an in vivo ligand for the Drosophila LAR receptor tyrosine phosphatase. *Curr. Biol*. **15,** 1701–1711.
25. O'Grady, P., Thai, T.C., and Saito, H. (1998) The laminin-nidogen complex is a ligand for a specific splice isoform of the transmembrane protein tyrosine phosphatase LAR. *J. Cell Biol*. **141,** 1675–1684.

**This article should be cited as follows:**

Rousselle, P. and Letourneur, F. (2009) Mysterious tasks of tyrosines in syndecan-1 cytoplasmic tail. *TheScientificWorldJOURNAL* **9**, 629–632. DOI 10.1100/tsw.2009.87.
